# LLIN evaluation in Uganda project (LLINEUP2): association between housing construction and malaria burden in 32 districts

**DOI:** 10.1186/s12936-024-05012-y

**Published:** 2024-06-17

**Authors:** Samuel Gonahasa, Martha Nassali, Catherine Maiteki‑Sebuguzi, Jane F. Namuganga, Jimmy Opigo, Isaiah Nabende, Jaffer Okiring, Adrienne Epstein, Katherine Snyman, Joaniter I. Nankabirwa, Moses R. Kamya, Grant Dorsey, Sarah G. Staedke

**Affiliations:** 1 Infectious Diseases Research Collaboration, Kampala, Uganda; 2 National Malaria Control Division, Ministry of Health, Kampala, Uganda; 3 Department of Medicine, University of California San Francisco, San Francisco, USA; 4 Department of Global Health and Development, London School of Hygiene & Tropical Medicine, London, UK; 5 Department of Clinical Research, London School of Hygiene & Tropical Medicine, London, UK; 6 Department of Medicine, Makerere University, Kampala, Uganda; 7 Department of Vector Biology, Liverpool School of Tropical Medicine, Liverpool, UK

**Keywords:** Malaria, House construction, Modern housing, Malaria incidence, Parasite prevalence, Uganda

## Abstract

**Background:**

Well-built housing limits mosquito entry and can reduce malaria transmission. The association between community-level housing and malaria burden in Uganda was assessed using data from randomly selected households near 64 health facilities in 32 districts.

**Methods:**

Houses were classified as ‘improved’ (synthetic walls and roofs, eaves closed or absent) or ‘less-improved’ (all other construction). Associations between housing and parasitaemia were made using mixed effects logistic regression (individual-level) and multivariable fractional response logistic regression (community-level), and between housing and malaria incidence using multivariable Poisson regression.

**Results:**

Between November 2021 and March 2022, 4.893 children aged 2–10 years were enrolled from 3.518 houses; of these, 1.389 (39.5%) were classified as improved. Children living in improved houses had 58% lower odds (adjusted odds ratio = 0.42, 95% CI 0.33–0.53, p < 0.0001) of parasitaemia than children living in less-improved houses. Communities with > 67% of houses improved had a 63% lower parasite prevalence (adjusted prevalence ratio 0.37, 95% CI 0.19–0.70, p < 0.0021) and 60% lower malaria incidence (adjusted incidence rate ratio 0.40, 95% CI 0.36–0.44, p < 0.0001) compared to communities with < 39% of houses improved.

**Conclusions:**

Improved housing was strongly associated with lower malaria burden across a range of settings in Uganda and should be utilized for malaria control.

## Background

In 2020, following twenty years of progress on malaria control, the downward trends in global malaria case incidence and malaria-related mortality reversed and have stalled, mostly driven by increased burden in African countries [1]. Vector control strategies, including long-lasting insecticidal nets (LLINs) and targeted use of indoor residual spraying (IRS), have been the foundation of malaria control in Africa, contributing to a 40% reduction in the incidence of malaria between 2000 and 2015 [2]. The recent plateau in malaria control gains has been attributed to inadequate coverage with existing control tools [3], COVID-19-related disruptions to health services, and gaps in funding for malaria control and research [4]. Emerging parasite resistance to artemisinin drugs in East Africa [5], and widespread resistance to pyrethroids and other insecticides [6], are other major threats. New tools are needed to accelerate progress on malaria control in Africa.

The association between housing quality and malaria has long been recognized [7]. The major malaria vectors in Africa, including *Anopheles gambiae *sensu lato (*s.l*.) and *Anopheles funestus*, prefer to feed at night when humans are indoors [8]. Thus, most malaria transmission occurs within the home. Traditional African houses are constructed with mud walls, thatch roofs and open eaves (the gap between the roof and the top of the wall), which serve as a primary entry point for *An gambiae s.l* [9]. Well-built housing limits entry of mosquito vectors and can reduce exposure to infectious bites. Temperatures inside houses with metal roofs may be higher than inside those with traditional thatch roofs, which can limit parasite development and reduce mosquito survival [10]. However, the heat inside houses with metal roofs may also discourage residents from using LLINs.

Historically, high-quality housing was an important strategy for improving public health and controlling malaria, but housing construction was overshadowed as IRS, and later LLINs, became key vector control tools [11]. Confronted by the intractable malaria burden in Africa and escalating insecticide resistance, housing construction is again gaining momentum as a malaria control tool. Recent studies have demonstrated that houses with improved construction are associated with reduced risk of malaria in individuals and households [12–14], but little evidence is available on the community-level impact of better housing and malaria burden. Moreover, the definition of ‘improved housing’ has not been standardized and varies between studies. To better understand associations between housing construction and malaria burden at the community-level, data from cross-sectional surveys and enhanced health facility-based surveillance conducted in 64 communities in 32 districts across Uganda were analyzed. House type was classified as ‘improved’ vs ‘not improved’, and communities were categorized according to the proportion of houses with improved construction, to test the hypothesis that the higher the proportion of improved houses in the community, the lower the burden of malaria.

## Methods

### Study sites

This study was embedded within a larger cluster randomized trial (LLINEUP2) designed to compare two types of newer generation LLINs distributed in the context of Uganda’s 2020–21 national LLIN distribution campaign. The 32 districts were selected using the following criteria: (1) not receiving IRS, (2) assigned by the Ministry of Health’s National Malaria Control Division to receive LLINs with piperonyl butoxide, and (3) high malaria transmission intensity.

### Health facility-based surveillance

Within each district, the Uganda Malaria Surveillance Project (UMSP) established enhanced malaria surveillance in two government-run health facilities, referred to as Malaria Reference Centers (MRCs); 64 MRCs from 32 districts were included. MRCs are level III/IV health facilities with functioning laboratories that provide care for appoximately 1000–3000 outpatients per month. At each MRC, individual-level data from standardized Health Management Information System (HMIS) outpatient registers are entered into an electronic database by on-site data officers. Patient age and village of residence and whether malaria was suspected are captured, along with information on diagnostic testing for malaria, if done, including the type of diagnostic test (microscopy or rapid diagnostic test [RDT]), and the test result (positive or negative). UMSP supports the sites with staff training, supervision, and laboratory supplies, as needed. Full-time regional surveillance assistants, each responsible for 8–10 MRCs, provide refresher training on malaria case management, review data quality, and perform external quality control for malaria microscopy, on a regular basis.

### Identification, enumeration, and mapping of target areas around each MRC

Target areas were identified around each MRC based on the assumption that most patients living in these areas would seek care at the MRC if they developed malaria. Target areas include the village surrounding the MRCs and adjacent villages, varying in size from 1 to 7 villages. Adjacent villages were included if: (1) they did not contain another health facility, (2) were in the same sub-county as the MRC, and (3) had a similar malaria burden as the MRC’s village. Using a map of the boundaries of the MRC target areas, study personnel systematically covered the entire area within the boundaries to enumerate and map all households using hand-held GPS devices. A household was defined as any single permanent or semi-permanent dwelling structure acting as the primary residence for a person or group of people that generally cook and eat together. This household enumeration list was used to generate the sampling frame for the cross-sectional surveys.

### Cross-sectional surveys

Between November 2021 and March 2022, cross-sectional community surveys were conducted within the 64 MRC target areas. Households randomly selected from the enumeration lists were approached for recruitment in each target area, until 50 households with at least one child aged 2–10 years were enrolled. Households meeting the following selection criteria were enrolled: (1) house occupied with at least one adult (≥ 18 years) present, who was (2) a usual resident who slept in the household on the night before the survey, and (3) agreed to provide written informed consent to participate in the survey. Households with no adult present were visited on at least three separate occasions before exclusion. At enrolled households, a standardized questionnaire was administered to the household head or their designate to collect information on demographics, bed net ownership and use, characteristics of house construction, and indicators of wealth. Children aged 2–10 years were invited to participate in a clinical survey. If consent was obtained, blood was collected by finger prick to prepare thick blood smears. Slides were stained with 2% Giemsa for 30 min and read by experienced laboratory technologists. A thick blood smear was considered negative when the examination of 100 high power fields did not reveal asexual parasites. For quality control, all slides were read by a second microscopist and a third reviewer settled discrepant readings.

### Statistical analysis

Data were analysed using Stata version 14.1 (College Station, TX), and R software. The exposure of interest was house type, defined using a previously-established binary classification system [15]. Houses were classified as ‘improved’ if they had all of the following: (1) walls made with synthetic materials (plaster, cement, iron sheets, or wood); (2) a synthetic roof (iron sheets, tiles); and (3) closed or absent eaves. All other houses were classified as ‘less-improved’. To determine community-level housing construction, the proportion of surveyed houses within the MRC target areas that were classified as improved was calculated and stratified into quartiles. After visual inspection of data (Fig. 2), showing the community-level proportion of houses with improved housing (x-axis) vs parasite prevalence among children aged 2–10 years (y-axis), and considering the lack of difference between the first two quartiles of community-level housing construction and their associations with the outcomes of interest, the first and second quartiles were combined, when assessing associations between community level measures of housing quality and malaria outcomes. Community-level housing construction within the MRC target areas was stratified into three categories: low-medium (1st and 2nd quartile; < 39% of houses classified as improved), medium–high (3rd quartile; 39–67% of houses improved), and high (4th quartile; > 67% of houses improved).

Outcomes of interest included: (1) individual-level parasitaemia among children aged 2–10 years, (2) community-level parasite prevalence (children aged 2–10 years), and (3) community-level malaria incidence (all ages). Community-level malaria incidence was defined as the total number of laboratory-confirmed cases of malaria diagnosed at the MRC among patients residing within the target area divided by the total person-time observed for the population of the target areas during the month of the cross-sectional survey.

For individual-level analyses, other covariates of interest included household wealth, adequate household coverage of LLINs (defined as one LLIN for every two household residents), and the child’s age and gender. Principal component analysis was used to generate a wealth index based on ownership of common household items, excluding variables used to define house type. Households were ranked by wealth scores and grouped into tertiles to provide a categorical measure of socioeconomic status, as done previously [16, 17]. Community-level analyses included mean household wealth index, mean age of community residents, proportion of the target area that was female, proportion of households with adequate LLINs, and an indicator variable representing the calendar month when the cross-sectional survey was done. Additional community-level covariates of interests including monthly precipitation [18] and enhanced vegetation index [19] (EVI; both lagged 1 month), presence of night time lights [20], distance to water, distance to roads [21], slope, and elevation [22] were generated from remotely sensed data measured as the mean within the target areas [23], calculated using the *exactextractr* package in R v3.5 [24].

Associations between house type and individual-level parasitaemia were estimated using a mixed effects logistic regression model with a random effect at the level of the household and adjustment for covariates of interest. Visual inspections of correlations between community-level housing and community-level outcomes were made using lowess smoothing. Associations between community-level housing and community-level outcomes were estimated using multivariable fractional response logistic regression (for parasite prevalence) and multivariable Poisson regression (for malaria incidence), adjusting for community-level covariates with precipitation and EVI included as non-linear terms using restricted cubic splines. For the community-level incidence model, the outcome was a count of laboratory confirmed malaria cases with an offset for the person-time in the target area. Individual-level measures of association were expressed as an odds ratio (OR) and community-level measures of association were expressed as a prevalence ratio (PR) or incidence rate ratio (IRR).

## Results

### Characteristics of residents and households

**Fig. 1:**
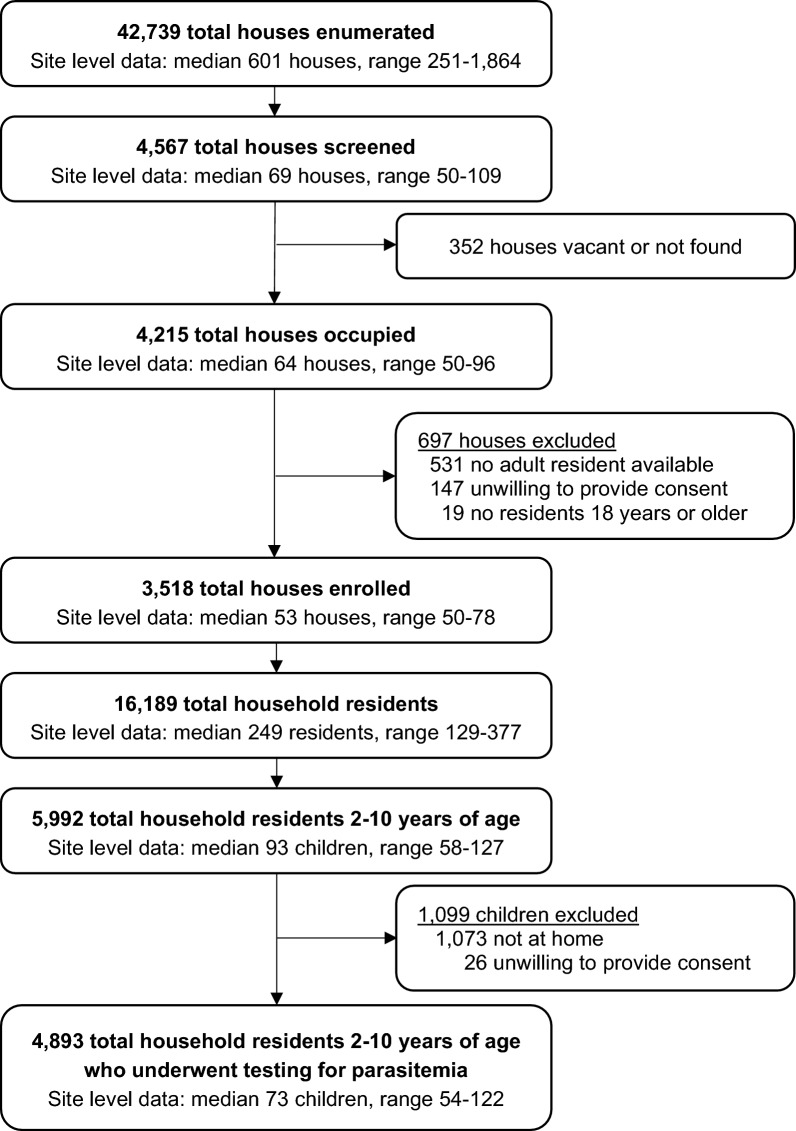
Trial Profile

A total of 42,739 households were enumerated across the 64 MRC target areas, 4215 occupied households were approached for recruitment, of which 3518 were enrolled (Fig. 1). The primary reasons households were excluded were the inability to locate an adult resident (531/697, 76.2%) and unwillingness to provide consent (147/697, 21.1%). Among 3518 households enrolled, a total of 16,189 residents were identified. Among 5992 residents aged 2–10 years, 4893 (81.7%) had blood smear results and were included in the analyses of parasitaemia, with a median of 73 children per target area. The primary reason residents 2–10 years of age were not included was absence from home on the day of the survey (1073/1099, 97.6%).

### Housing characteristics

Of 3518 households enrolled, 1903 (54.1%) had synthetic walls, 2010 (57.1%) had synthetic roofs, and 2197 (62.5%) had closed or absent eaves. Considering all three characteristics together, 1389 (39.5%) houses were classified as improved. Of these, almost all (1348/1389, 97.0%) were constructed with walls made of cement or bricks covered with plaster, an iron sheet roof, and closed or absent eaves (Table 1). In contrast, less-improved houses (n = 2219) had much greater variability in design, commonly constructed with brick walls, thatch roof, and open eaves (519, 24.4%), or walls made of mud and poles, thatch roof, and open eaves (260, 12.2%).

**Table 1:** Classification of house type based on specific components using in construction

House type variable	Materials used for walls	Materials used for roof	Eaves	Frequency (%)
Improved (N = 1389)	Bricks with plaster or cement	Iron sheets	Closed or absent	1348 (97·0%)
	Cement blocks	Iron sheets	Closed or absent	20 (1·4%)
	Bricks with plaster or cement	Cement	Closed or absent	8 (0·6%)
	Iron sheets	Iron sheets	Closed or absent	6 (0·4%)
	Bricks with plaster or cement	Tiles	Closed or absent	3 (0·2%)
	Bricks with plaster or cement	Asbestos	Closed or absent	2 (0·1%)
	Wood	Iron sheets	Closed or absent	2 (0·1%)
Less improved (N = 2129)	Bricks alone	Thatched	Open	519 (24·4%)
	Mud and poles	Thatched	Open	260 (12·2%)
	Bricks with plaster or cement	Iron sheets	Open	231 (10·9%)
	Mud and poles	Thatched	Closed or absent	230 (10·8%)
	Bricks alone	Thatched	Closed or absent	179 (8·4%)
	Bricks alone	Iron sheets	Closed or absent	158 (7·4%)
	Bricks with plaster or cement	Thatched	Open	141 (6·6%)
	Bricks with plaster or cement	Thatched	Closed or absent	132 (6·2%)
	Mud and poles	Iron sheets	Closed or absent	85 (4·0%)
	Bricks alone	Iron sheets	Open	73 (3·4%)
	Mud and poles	Iron sheets	Open	69 (3·2%)
	Other miscellaneous combinations			52 (2·4%)

### Association between house type and parasitaemia among children 2–10 years of age

Among 4,893 children aged 2–10 years tested by microscopy, 1,175 (24.0%) were positive for asexual parasites. Parasite prevalence was 14.7% (268/1,827) for children living in improved houses compared to 29.6% (907/3066) for children living in less-improved houses classified. In the multivariate analysis, children living in improved houses classified had a 58% lower odds (odds ratio 0.42, 95% CI 0.33–0.53, p < 0.0001) of parasitaemia compared to children living in less-improved houses (Table 2). Other factors independently associated with a lower odds of parasitaemia included greater household wealth, decreasing age, female gender, and living in a house with adequate LLIN coverage (one LLIN for every two household residents).

**Table 2:** Individual or household level factors associated with parasitaemia among children 2–10 years of age

Variable	Category	Parasitaemia, n/N (%)	Univariate ^c^		Multivariate ^c^	
			OR (95% CI)	p-value	OR (95% CI)	p-value
House type ^a^	Less improved	907/3066 (29·6%)	Reference group		Reference group	
	Improved	268/1827 (14·7%)	0·32 (0·26–0·40)	< 0·0001	0·42 (0·33–0·53)	< 0·0001
Household wealth	Poorest	521/1639 (31·8%)	Reference group		Reference group	
	Middle	416/1668 (24·9%)	0·62 (0·50–0·77)	< 0·0001	0·72 (0·58–0·90)	0·0042
	Least poor	238/1586 (15·0%)	0·28 (0·22–0·36)	< 0·0001	0·42 (0·32–0·55)	< 0·0001
Age categories in years	8–10	264/838 (31·5%)	Reference group		Reference group	
	6–7	378/1336 (28·3%)	0·82 (0·64–1·05)	0·12	0·75 (0·59–0·96)	0·024
	4–5	281/1277 (22·0%)	0·50 (0·39–0·65)	< 0·0001	0·45 (0·35–059)	< 0·0001
	2–3	252/1442 (17·5%)	0·36 (0·27–0·47)	< 0·0001	0·31 (0·24–0·41)	< 0·0001
Gender	Male	618/2369 (26·1%)	Reference group		Reference group	
	Female	557/2524 (22·1%)	0·76 (0·64–0·90)	0·0014	0·74 (0·62–0·87)	0·0004
Lives in household with adequate number of LLINs ^b^	No	611/2302 (26·5%)	Reference group		Reference group	
	Yes	564/2591 (21·8%)	0·72 (0·60–0·87)	0·0006	0·79 (0·65–0·95)	0·013

^a^Improved houses defined as those with closed eaves and synthetic materials used for walls and roof; all other houses defined as less improved

^b^Defined as least 1 LLIN per 2 household members

^c^Adjusted for repeated measures from the same household

### Associations between community-level measure of housing and malaria outcomes

**Fig. 2:**
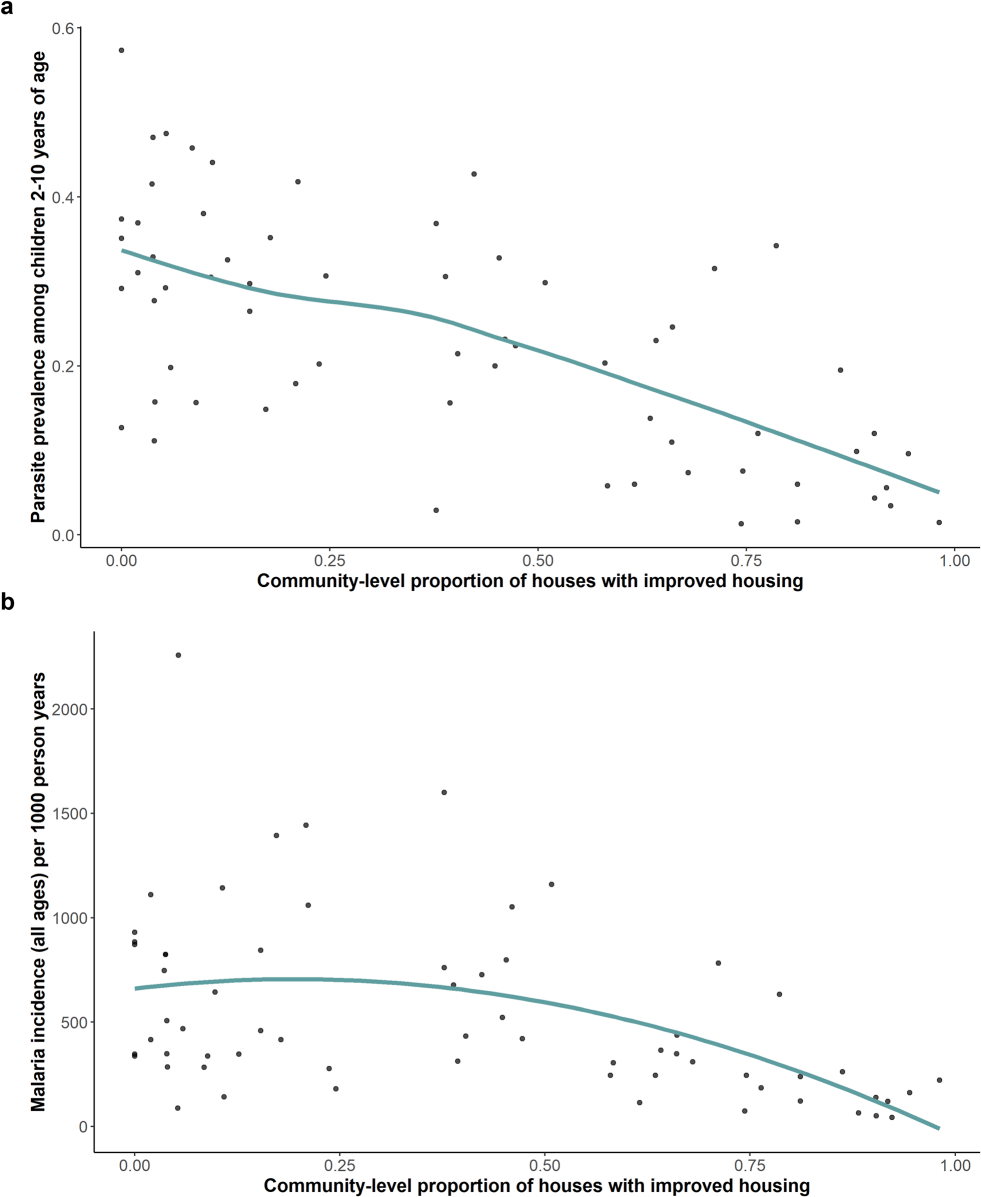
Community level impact of improved housing on malaria prevalence (in children aged 2–10 years) and incidence (all ages)

**Fig. 3:**
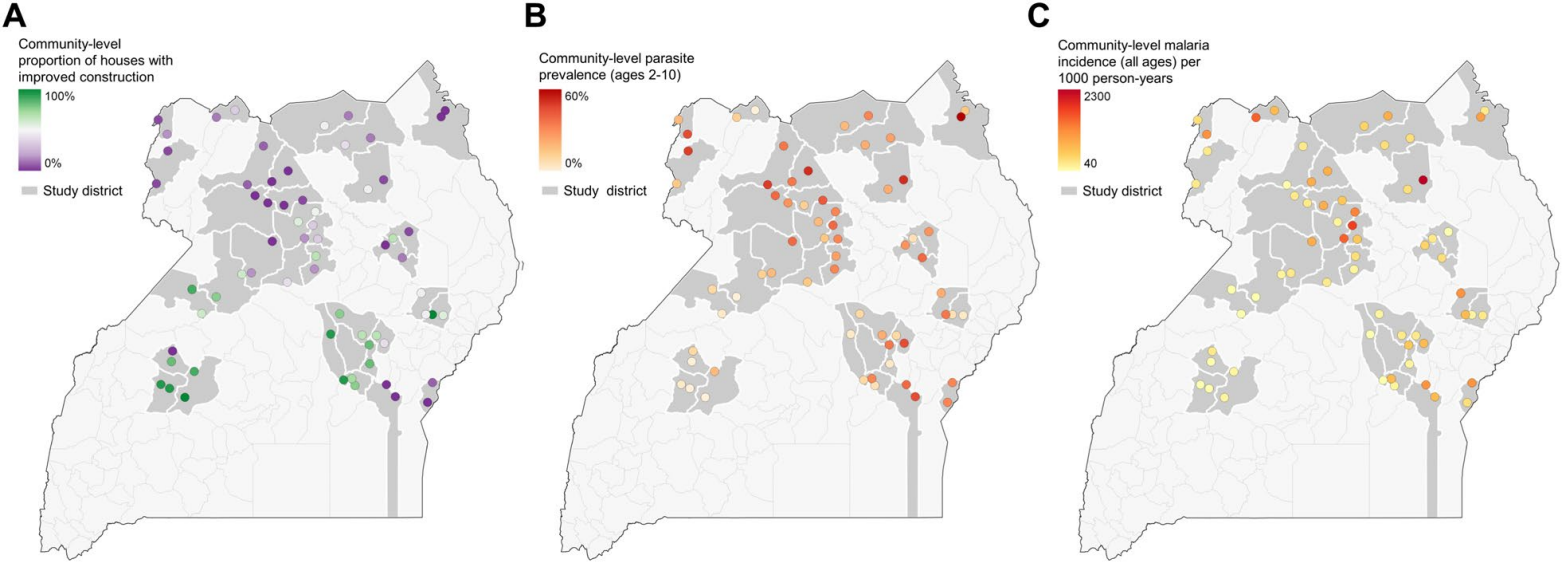
Maps showing the geographic distribution of housing and malaria indices across the study area

Community-level measures of housing varied widely with the proportion of houses classified as improved ranging from 0% to 98.1% (median 38.3%, IQR 7.2–67.1%) across the 64 sites. Community-level measures of parasite prevalence ranged from 1.3 to 57.4% (median 22.7%, IQR 11.6–32.8%) and malaria incidence ranged from 42 to 2258 episodes per 1000 person years (median 390, IQR 244–790). The community-level proportion of houses that were classified as improved was inversely related to parasite prevalence and malaria incidence, with both indicators decreasing as the proportion of houses that were improved increased within communities. This was particularly true when the community-level proportion of improved houses exceeded ~ 40% (Fig. 2). Geographic clustering of community-level measures of housing and malaria burden was also observed (Fig. 3). Sites in central Uganda tended to have the highest proportion of improved houses and the lowest measures of parasite prevalence and malaria incidence, while the reverse was true for sites in the northern and south-eastern parts of the country.

As the proportion of housing classified as improved in communities increased, parasite prevalence and malaria incidence were lower (Table 3). In communities with low-medium improved housing (1st and 2nd quartile, < 39% of houses), mean parasite prevalence was 30.5% (SD 12.4%) and mean malaria incidence was 705 episodes per 1000 person-years (SD 488), while in communities with a high proportion of improved houses (4th quartile, > 67%), mean parasite prevalence was 10.4% (SD 10.0%) and mean malaria incidence was 228 episodes per 1000 person-years (SD 205). In the multivariate analysis controlling for community-level measures of wealth, precipitation, vegetation, night lights, distance to water, distance to roads, slope and elevation, communities with a high proportion of houses classified as improved had a 63% lower parasite prevalence (prevalence rate 0.37, 95% CI 0.19–0.70, p < 0.0021) and 60% lower incidence of malaria incidence (incidence rate ratio 0.40, 95% CI 0.36–0.44, p < 0.0001) compared to communities with a low-medium proportion of improved houses (Table 3).

**Table 3:** Associations between community-level proportion of houses with improved construction and malaria outcomes

Proportion of houses with improved construction	Number of sites	Mean parasite prevalence^a^	Unadjusted		Adjusted ^b^		Mean malaria incidence ^c^	Unadjusted		Adjusted ^b^	
			PR (95% CI)	p-value	PR (95% CI)	p-value		IRR (95% CI)	p-value	IRR (95% CI)	p-value
Low-medium (< 39%)	32	30.5%	Reference group		Reference group		705	Reference group		Reference group	
Medium–high (39–67%)	16	21.4%	0·62 (0·44–0·88)	0·0067	0·63 (0·45–0·89)	0·0086	510	0·76 (0·72–0·80)	< 0·0001	0·68 (0·63–0·73)	< 0·0001
High (> 67%)	16	10·4%	0·27 (0·15–0·46)	< 0·0001	0·37 (0·19–0·70)	0·0021	228	0·34 (0·32–0·37)	< 0·0001	0·40 (0·36–0·44)	< 0·0001

^a^Proportion of children 2–10 years of age with a positive blood smear by microscopy

^b^Adjusted analysis includes community-level measures of wealth, precipitation, vegetation, night lights, distance to water, distance to roads, slope, elevation, the proportion of households with adequate LLINs, mean age, proportion female, and calendar month of measurement·

^c^Episodes of malaria (all ages) per 1000-person years

## Discussion

In sub-Saharan Africa, over 80% of malaria is transmitted indoors at night [25]. High-quality housing has been shown to limit entry of *Anopheles* vectors and can protect against malaria [12, 26]. The relationship between housing construction and malaria indicators in 64 communities across Uganda was explored. This study found that children living in houses with improved construction had a lower odds of malaria parasitaemia than those living in less-improved houses, providing additional evidence that well-built housing can reduce malaria risk for individual children. Greater household wealth, lower age, female gender, and adequate LLIN coverage were independently associated with a lower odds of parasitaemia in these children. The association between community-level housing construction and malaria indicators, a novel aspect of this study, was also explored. As the proportion of housing classified as improved increased within communities, parasite prevalence and malaria incidence decreased markedly. Further research is needed to understand the impact of specific features of house construction on malaria risk, alone and in combination with other malaria control interventions, to guide optimal design for initial house construction and retrofit modifications.

In this study, houses were classified as improved based on the construction of the walls (bricks with plaster, cement, metal, or wood), roof (iron sheet, tiles, cement, or asbestos) and eaves (closed or absent). Similar criteria have been used to define houses as modern in other studies [16, 27], while some studies have incorporated floor materials into the housing classification [13]. Rather than define houses as ‘modern’, the study team opted to classify houses as ‘improved’ vs ‘less-improved’, which the team felt captured the heterogeneity of housing from rural settings more appropriately. Interestingly, although the criteria for improved housing allowed for many scenarios, 97% of houses classified as improved were characterized by a specific house type with walls made of bricks covered with plaster or cement, roofs with iron sheets, and closed or absent eaves. Higher-quality housing is theorized to protect against malaria by providing a physical barrier and potentially raising temperatures inside houses [13]. In The Gambia and Malawi, entry of *Anopheles* mosquitoes was lower in houses with closed eaves [9, 28], and in Equatorial Guinea, parasite prevalence was lower in children living in houses with closed eaves and screened windows [29]. Higher temperatures in houses with metal roofs may also increase mosquito mortality [10], and the odds of malaria infection in residents of houses with metal roofs was lower than in residents of mud-roofed houses [30]. In the latest Cochrane systematic review of housing modifications to prevent malaria, trials conducted in sub-Saharan Africa between 2009 and 2022 evaluated house screening (of windows, doors, eaves, and ceilings—alone, or in combination), roof modifications, and installation of eave tubes [14]. House improvements were found to protect against anaemia and may reduce prevalence of malaria parasitaemia. Reduced indoor vector density was observed in some studies, and findings on malaria incidence were mixed [14].

In this study, adequate LLIN coverage was associated with lower odds of parasitaemia in children, as expected. A cross-sectional analysis of data from 21 African countries collected in nationwide surveys between 2008 and 2015 suggested that improved housing (brick or concrete walls and metal roof) lowered the odds of malaria infection in children by 9–14%, which was similar to the 15–16% reduction in odds provided by LLINs [13]. The community-level benefits of LLINs when high coverage is achieved are well-described [31, 32], and have underpinned the strategy of mass distribution of LLINs to achieve universal coverage [33]. However, from available literature, this is the first study to demonstrate the benefits of improved housing on malaria burden within communities. This study found that community-level house construction varied widely across Uganda and clustered geographically. In addition, increased household wealth was independently associated with a lower odds of malaria parasitaemia in individual children. The link between malaria and poverty is well-recognized, but the causal mechanisms for this association are not entirely clear [34, 35]. House construction and food security have been suggested as possible mediators in the relationship between malaria and poverty [35]. House design is changing rapidly in Uganda [16], and elsewhere in Africa, with the proportion of housing defined as improved (with improved water and sanitation, adequate living area and durable construction) in sub-Saharan Africa increasing from 11% in 2000 to 23% in 2015 [36]. The population of Africa is expected to double by 2050 [37], and the continent is facing substantial economic growth and urbanization, presenting an exceptional opportunity to build homes that reduce mosquito entry, while meeting the increased demand for housing [13, 16].

This study had several limitations. First, it utilized an observational study design which limits the ability to determine causality. Although randomized controlled trials remain the gold standard for evaluating the impact of interventions, implementing housing modifications on the scale assessed in this research study would have been financially and logistically prohibitive. Second, parasite prevalence was measured cross-sectionally over a period of five months, and community-level malaria incidence was measured only during the month of the cross-sectional survey, so the results could have been affected by seasonality of malaria transmission or other environmental factors. However, the community-level analyses controlled for the calendar month of the cross-sectional survey, monthly precipitation, and enhanced vegetation index. Third, while poorly fitting doors and windows could provide entry points for mosquitoes, information on the construction of doors and windows, and whether they were well-fitted, was not systematically captured and cannot be included in the classification of housing construction. Fourth, MRCs were selected using convenience sampling and may not have been representative of other MRCs in the district. However, the results of this study contribute to evidence suggesting that incremental improvements in housing design, implemented during initial construction or through retrofit modifications, could have a significant impact on malaria burden.

## Conclusions

In this study, as the proportion of houses classified as improved within communities increased, parasite prevalence and malaria incidence fell. This study demonstrates an association between improved housing and lower malaria burden at the community level, across a wide range of settings in Uganda. This demonstrates that improved housing construction, specifically synthetic roofs and walls combined with closed or absent eaves, can reduce malaria burden in individual children and communities. These results support the existing literature demonstrating that well-built houses protect individual children against malaria, while adding new evidence that housing construction provides protection at the community level. Improved housing is an underutilized tool in the fight against malaria. With the stalled progress on malaria burden in Africa, and the looming challenges of insecticide and artemisinin resistance, housing construction should be seriously considered as a non-insecticidal control intervention. Improved housing could complement LLINs and IRS in malaria control efforts, while contributing holistically to overall public health.

## Data Availability

De-identified data collected from the cross-sectional surveys and a data dictionary defining each field in the datasets will be made publicly available at the time of publication through ClinEpiDB (https://clinepidb.org/ce/app).
